# AI-Assisted Differentiation of Dengue and Chikungunya Using Big, Imbalanced Epidemiological Data

**DOI:** 10.3390/tropicalmed11020040

**Published:** 2026-01-30

**Authors:** Thanh Huy Nguyen, Nguyen Quoc Khanh Le

**Affiliations:** 1 International Ph.D. Program in Medicine, College of Medicine, Taipei Medical University, Taipei 110301, Taiwan; 2 In-Service Master Program in Artificial Intelligence in Medicine, College of Medicine, Taipei Medical University, Taipei 110301, Taiwan; 3 AIBioMed Research Group, Taipei Medical University, Taipei 110301, Taiwan; 4 Translational Imaging Research Center, Taipei Medical University Hospital, Taipei 110301, Taiwan

**Keywords:** dengue, chikungunya, machine learning, deep learning, neglected tropical diseases, arboviral infections, diagnosis, imbalanced data, public health surveillance

## Abstract

Dengue and chikungunya are endemic arboviral diseases in many low- and middle-income countries, often co-circulating and presenting with overlapping symptoms that hinder early diagnosis. Timely differentiation is critical, especially in resource-limited settings where laboratory testing is unavailable. We developed and evaluated machine-learning (ML)- and deep-learning (DL) models to classify dengue, chikungunya, and discarded cases using a large-scale, real-world dataset of over 6.7 million entries from Brazil (2013–2020). After applying the Synthetic Minority Oversampling Technique (SMOTE) to address class imbalance, we trained six ML models and one artificial neural network (ANN) using only demographic, clinical, and comorbidity features. The Random Forest model achieved strong multi-class classification performance (Recall: 0.9288, the Area Under the Curve (AUC): 0.9865). The ANN model excelled in identifying chikungunya cases (Recall: 0.9986, AUC: 0.9283), suggesting its suitability for rapid screening. External validation confirmed the generalizability of our models, particularly for distinguishing discarded cases. Our models demonstrate high-accuracy in differentiating dengue and chikungunya using routinely collected clinical and epidemiological data. This work supports the development of Artificial Intelligence-powered decision-support tools to assist frontline healthcare workers in under-resourced settings and aligns with the One Health approach to improving surveillance and diagnosis of neglected tropical diseases.

## 1. Introduction

Neglected tropical diseases (NTDs) continue to burden impoverished communities in tropical and subtropical regions, contributing significantly to global health inequities. Among these, arboviral infections such as dengue and chikungunya have re-emerged as major public health concerns [1], particularly in low- and middle-income countries across Latin America, Southeast Asia, and Africa [2,3]. Both diseases are transmitted by the *Aedes* mosquito and frequently co-circulate, making clinical differentiation challenging due to their overlapping symptomatology [4].

In 2021, the World Health Organization (WHO) declared the roadmap for NTDs in 2021–2030 with the new ambitious target: reducing 75% of deaths from vector-borne NTDs, including dengue and chikungunya [5]. However, the global scenario of dengue is still not optimistic: over 6.5 million cases and more than 7300 dengue-related deaths were reported across all six WHO regions in 2023, with the Americas reporting the highest number of infected individuals with 4.5 million cases and 2300 deaths. The year 2024 surpassed 2023 as the worst year for recorded dengue cases, with over 10 million cases reported, 24,000 severe cases, and 6508 deaths [6]. Dengue cases increased more than 50%, from 26.5 million in 1990 to 59.0 million in 2021 [7], with a higher incidence in children and adolescents [8].

Although dengue usually presents with common flu-like symptoms such as fever, myalgia, and headache, and most cases self-recover, a small number of patients develop severe dengue [9]. Regardless of if a patient has prior exposure to only dengue or combined with another arboviral viruses, such as Zika, they are at an increased risk of developing severe dengue, which could be fatal [10].

Beside dengue, chikungunya is also a public health concern as a re-emerging vector-borne disease [11] with 18.7 million cases in 110 countries between 2011 and 2020, causing 1.95 million disability-adjusted life years, USD 2.8 billion in direct costs, and USD 47.1 billion in indirect costs worldwide; the majority of the disease burden was observed in the Americas [12,13]. An estimated 51% of symptomatic chikungunya patients with laboratory-confirmed tests developed chronic disability after infection [14]. The clinical manifestations of chikungunya at the early stage include fever, arthralgia, myalgia, and joint pain and swelling, which are almost indistinguishable from dengue and other febrile diseases [15].

Many health efforts have been made to differentiate dengue from chikungunya and other febrile diseases. A three-year study in Puerto Rico identified arthritis, joint pain, skin rash, any bleeding, and irritability as clinical predictors that distinguish chikungunya from dengue; while joint pain, muscle, bone, or back pain, skin rash, and red conjunctiva are significant predictors for chikungunya compared with other acute febrile infections [16]. Researchers in Brazil proposed a clinical rule scoring system to diagnose chikungunya infection in a dengue-endemic area, using fever, exanthema, myalgia, arthralgia or arthritis, and joint edema; this system achieved an AUC of 0.695 [17]. However, in remote areas, human- and equipment resources are limited. While dengue and chikungunya share the same vector—known as the Aedes mosquito—similar symptoms with other arboviral pathogens make correctly diagnosing patients more challenging [18,19,20]. Therefore, an alternative approach is needed to assist healthcare workers in the early detection and classification of patients with these diseases without laboratory confirmation.

Machine Learning (ML), a subset of Artificial Intelligence (AI), is algorithms that can learn the patterns inside a dataset and use these experiences to automatically improve the accuracy of their output [21]. In recent years, advances in clinical practice using ML and its subfield—Deep Learning (DL)—have proven to improve diagnostic performance [22,23,24], including arboviral-diseases detection [25,26]. These techniques can assist in classifying, supporting diagnosis and treatment for patients with arboviral diseases in resource-limited settings. Previous studies attempted to develop ML models for a binary classification of dengue fever (DF). These models aimed to predict dengue positivity/negativity or to differentiate between severe dengue (SD) and non-severe dengue, using various types of data, including meteorology, genomic, socio-demographic, clinical, and laboratory data [27]. Recently, a novel approach using micro-spectroscopy techniques combined with machine learning showed a promising application in rapid classification of dengue and chikungunya in remote areas [28].

Due to the aim of predicting clinical dengue cases, we examined the previous literature that applied ML for dengue diagnosis based on demographic, clinical, and laboratory data only, focusing on dataset size, features used, the ML models implemented, and the metrics used to evaluate the models’ performance. For example, Ho et al. applied different ML models to identify 2942 dengue cases from a dataset of 4894 patients with dengue-like illnesses, using only age, body temperature, white blood cell count, and platelet count as input features. Logistic regression (LR), Decision Tree (DT), and Deep Neural Networks (DNN) were used to build predictive models; the proposed DL model achieved the best performance with an AUC of 0.8587 [29]. Abdualgalil et al. analyzed 6694 samples containing one continuous variable (age) and 20 binary categorical features (including sex, fever, headache, arthralgia, myalgia, conjunctivitis, skin rash, generalized weakness, jaundice, decrease in urine or anuria, abdominal pain, vomiting,) with the train/test split ratio of 70/30 to build five ML models: k-Nearest Neighbor (KNN), Gradient Boosting Classifier, eXtreme Gradient Boosting (XG), Extra Tree Classifier (ETC), and Light Gradient Boosting Machine [30]. The target variable was dengue positivity or negativity; the ETC model using the hold-out cross-validation approach achieved the highest accuracy of 0.9912.

In predicting SD cases, Phakhounthong et al. used DT to predict 38 SD cases out of 198 laboratory-confirmed dengue cases in Cambodian children, using five clinical and laboratory attributes (hematocrit, Glasgow Coma Score, urinary protein, creatinine, and platelet count). The DT model achieved 0.605 sensitivity, 0.65 specificity, and 0.641 accuracy [31]. Huang et al. analyzed 798 laboratory-confirmed dengue cases, including 138 SD cases, to develop various ML models for assessing the risk of dengue severity based on six features: age, sex, viral RNA amounts, the positivity of NS1, and IgM and IgG test results. Different ML methods, including LR, Random Forest (RF), Gradient Boosting Machine (GB), Support Vector Classifier (SVC), and Artificial Neural Networks (ANN)—a DL algorithm—were applied for building prognostic models. The ANN model outperformed others with an AUC of 0.8324 and 0.7523 accuracy [32].

Besides binary classification models, the development of multi-class classification algorithms, which can differentiate between dengue and other mosquito-borne diseases, are of great importance to clinicians when more than one arbovirus is present in endemic areas. Lee et al. implemented LR and DT models to differentiate between 862 DF, 55 dengue hemorrhagic fever (DHF), and 117 chikungunya cases in two scenarios: with and without laboratory testing (suitable for well-resourced and resource-limited settings, respectively). Multiple demographic, epidemiological, and clinical features were used for the prediction. Without laboratory results, the DT model achieved an overall AUC of 0.59 in classifying DF and chikungunya cases, while performing better in discriminating DHF versus chikungunya with 0.91 AUC [33].

Tabosa de Oliveira et al. used seven ML models: RF, Adaptative Boosting (AD), GB, XG, KNN, NB, and Multilayer Perceptron, to train a dataset of 17,272 records, with 5724 for each of the three classes: dengue, chikungunya, and others (patients classified as “inconclusive” or “negative” for both dengue and chikungunya). The dataset consists of 26 features: socio-demographic (age, sex, gestational age in case sex is female, race, residence area, days that patient feels the symptoms), clinical (fever, myalgia, headache, rash, vomiting, nausea, back pain, conjunctivitis, arthritis, arthralgia, petechiae, tourniquet test, eye pain), and comorbidities (diabetes, hypertension, and hematological, liver, kidney, peptic acid, and autoimmune disease). The GB model achieved the best performance with 0.6240 accuracy, 0.6257 precision, 0.6205 recall, and 0.6196 F1-score [34].

Previous studies that focused on multi-class classification of dengue and other diseases are still limited compared with binary tasks [35]. Moreover, a previous study aimed to differentiate dengue with malaria, leptospirosis, and scrub typhus, also indicated that ML models (DT, RF, AD) only achieved 55–60% overall predictability on the multi-class classification task, far lower than binary classification using the LR model with an average of 79–84% correct predictions for one versus other diseases [36]. Previous studies have examined the applicability of ML-based models for disease diagnosis; however, there are no studies that have attempted to apply DL algorithms for multiclass classification of multiple arboviral diseases [35]. To the best of our knowledge, there are no studies that have investigated the multi-class task on an imbalanced dataset with more than 100,000 records.

Therefore, in this study, we built different ML and DL models to investigate the ability of differentiating dengue and chikungunya cases with discarded cases (inconclusive cases of the two diseases) using a big, highly imbalanced open-source dataset.

## 2. Materials and Methods

### 2.1. Data Collection

**Figure 1:**
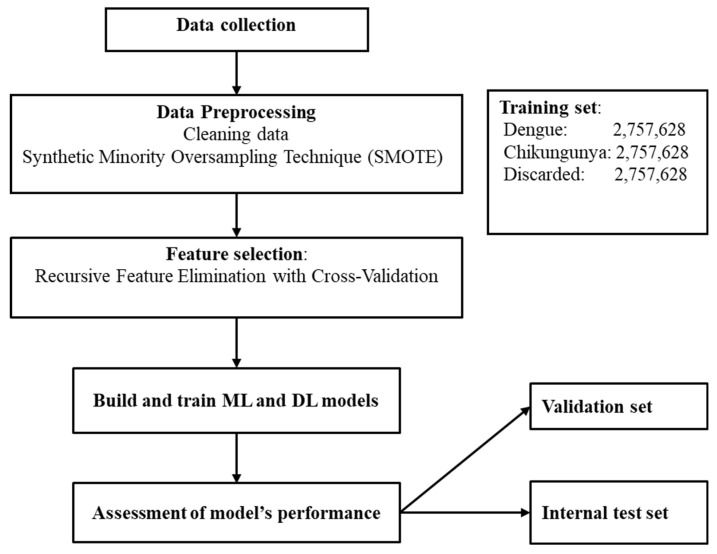
The workflow of this study.

We used an open-source dataset from da Silva Neto et al. [37], which consists of 4,307,513 dengue, 325,000 chikungunya, and 2,100,029 discarded cases in Brazil from 2013 to 2020 for classification. There are 55 variables, but 9 from laboratory data were excluded. The remaining features were classified into three groups: demographic, clinical, and comorbidity data. One important characteristic of this dataset is that some important diagnostic features such as days from symptom onset and severity markers were not collected by the researchers. Figure 1 illustrates the workflow of this study.

### 2.2. Data Preprocessing

First, the data was checked for missing values and typos. Next, the outcome variable was encoded as follows: 0 for discarded cases, 1 for chikungunya, and 2 for dengue. Apart from the age variable, which is numeric, all other categorical variables were converted into numeric format according to the number of classes within each variable. Third, the dataset was split into two parts: training data and testing data, with an 80/20 ratio. The testing data is also called the internal test set, which has similar features to the training set, including years, geographic regions, municipalities, and class distribution. This test set is kept separately during model training. Then, the training data was split further into two parts, with the same ratio for training and validation purposes. For the training dataset, the imbalanced data was handled using various techniques, including random undersampling, random oversampling, and the Synthetic Minority Oversampling Technique (SMOTE) [38], since the dataset used in this study has the minority class of interest (chikungunya cases). The performance of models was compared after applying different approaches to handle the issue of imbalanced data and SMOTE showed the prominent advantage over techniques. Therefore, SMOTE was applied in this study despite its potential concern of overfitting. After applying SMOTE for training data, we had 8,272,884 samples, and the number of instances in each class after using SMOTE was 2,757,628. Important features for predicting dengue and chikungunya were chosen after using the ML algorithm which shows the best performance as baseline. Last, train- and test sets were normalized using z-score scaling technique.

### 2.3. ML Model Development

We used various ML techniques to develop six models to diagnose different diseases: Random Forest (RF), Decision Tree (DT), Adaptive Boosting (AD), Gradient Boosting Machine (GB), eXtreme Gradient Boosting (XG), and K-nearest Neighbor (KNN). The proposed algorithms were trained with different hyperparameters and random_state = 42, using RandomizedSearchCV package in Scikit-Learn library. Table 1 displays the hyperparameters that presented the best results. The model with the best performance was chosen for feature selection with the Recursive Feature Elimination with Cross-Validation (RFECV) technique, using the following parameters: estimator = the ML classifier with best performance; step = 1; cv = StratifiedKFold (5); scoring = ‘accuracy’.

### 2.4. Artificial Neural Network (ANN) Model

The main advantage of ANN model is that it does not require human intervention for any of its processes, which allows automatic feature extraction in comparison with ML [39]. Table 2 describes model configuration of ANN model. The hidden layer had two layers with 64 output shapes.

The above model was trained in 30 epochs, 128 batch_size using keras and tensorflow package, and hyperparameter optimization was performed with RandomSearchCV to perform effective differential diagnosis between three classes.

### 2.5. Model Evaluation

In this research, we have evaluated our multi-class arbovirus diseases classification models by using the accuracy, precision, recall, specificity, balanced accuracy, F1-score, and area under the receiver operating characteristics (ROC) curve (AUC). These evaluation metrics are based on the confusion matrix, which seeks to calculate True Positive (TP), True Negative (TN), False Positive (FP), and False Negative (FN).

Accuracy measures the model’s performance according to the total samples correctly classified, and is defined as

(1) $$\mathrm{Accuracy}=\frac{\left(TP+TN\right)}{\left(TP+FP+TN+FN\right)}$$

Precision, also called positive predictive value, is the metric used to represent the proportion of positive classifications that are true positive, calculated as

(2) $$\mathrm{Precision}=\frac{TP}{\left(TP+FP\right)}$$

Recall, also called sensitivity, determines the proportion of real positives that were correctly classified, defined as

(3) $$\mathrm{Recall}=\frac{TP}{\left(TP+FN\right)}$$

Specificity determines the proportion of real negatives that were correctly classified, defined as

(4) $$\mathrm{Specificity}=\frac{TN}{TN+FP}$$

Balanced accuracy is the arithmetic mean of sensitivity and specificity, and is defined as

(5) $$\mathrm{Balanced}\;\mathrm{accuracy}=\frac{Sensitivity+Specificity}{2}$$

F1-score is a metric that calculates the harmonic mean of precision and recall, and is defined as

(6) $$F1\text{-}\mathrm{score}=2\;\times \;\frac{Precision\times Recall}{\left(Precision+Recall\right)}$$

The metrics for multi-class classification were obtained by using macro averaging. Initially, precision, recall (sensitivity), specificity, F1-score, accuracy, and balanced accuracy were calculated for each class. The metrics for multi-class classification were obtained using macro averaging, i.e., by taking the average of the values obtained for three classes. Balanced accuracy, along with AUC metric, are identified to be more robust to imbalanced data than traditional accuracy [40]. In multi-class classification task, balanced accuracy is defined as the average of recall, which equals the macro-average recall.

### 2.6. Software

Tensorflow and Keras frameworks implemented the proposed method. A free cloud service from Google Colab performed the training and testing process. The evaluations metrics were calculated using the Scikit-Learn library version 1.5.0.

## 3. Results

### 3.1. Data Characteristics

As depicted in Table 3, 55.8% of cases were women with a mean age of 33 years; most did not have information about race, stage in pregnancy, or education degree. The most common symptoms included fever (37.3%), headache (34.5%), myalgia (34%), retro-orbital pain (14.3%), and nausea (14.2%).

### 3.2. ML Models’ Performance

Table 4 presents a comparison of the performance of ML models with and without applying the SMOTE technique on training set. Before applying SMOTE, the XG model outperformed others with a recall of 0.9333, precision of 0.8711, F1-score 0.8983, and an AUC of 0.9831. Among the ML models after applying SMOTE, RF algorithm showed the best performance in classifying dengue, chikungunya, and discarded cases with the macro-average values of accuracy (0.9292), recall/balanced accuracy (0.9288), precision (0.9111), F1-score (0.9196), and AUC (0.9853).

**Figure 2:**
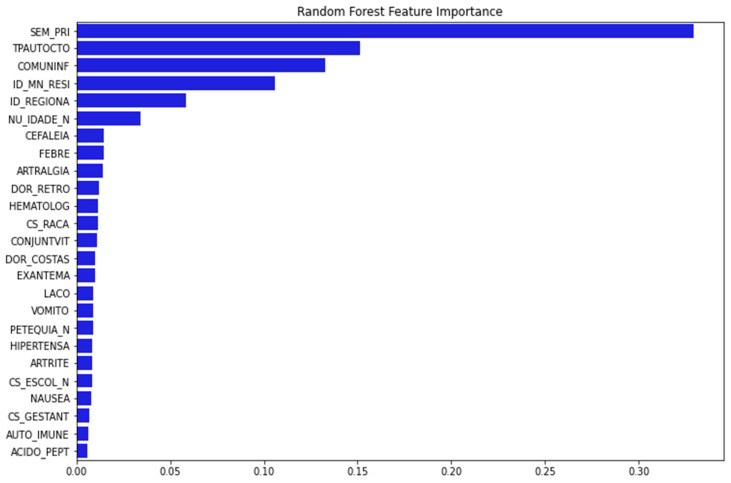
The important features are selected using RFECV technique with RF. The *y*-axis presents the 25 important features selected using RFECV technique (blue bar). The *x*-axis presented the percentage of importance. (Note: SEM_PRI: Epidemiological week of onset of symptom; TPAUTOCTO: Indicates whether the case is indigenous to the area of residence; COMUNINF: City where the patient was infected; ID_MN_RESI: City of the patient; ID_REGIONA: Health care regional code (where the health unit or other reporting source is located); NU_IDADE_N: Patient age; CEFALEIA: Headache; FEBRE: Fever; ARTRALGIA: Arthralgia; DOR_RETRO: Retro-orbital pain; HEMATOLOG: Hematological disease; CS_RACA: Patient Race; CONJUNTVIT: Conjunctivitis; DOR_COSTAS: Back Pain; EXANTEMA: Rash; LACO: Tourniquet test; VOMITO: Vomiting; PETEQUIA_N: Petechiae; HIPERTENSA: Hypertension; ARTRITE: Arthritis; CS_ESCOL_N: Patient education; NAUSEA: Nausea; CS_GESTANT: Gestational Age of the Patient (Quarter) in case Sex is Female; AUTO_IMMUNE: Autoimmune disease; ACIDO_PEPT: Peptic acid disease).

Figure 2 depicts 25 important variables chosen for model training after running the feature importance procedure with RF as baseline.

**Figure 3:**
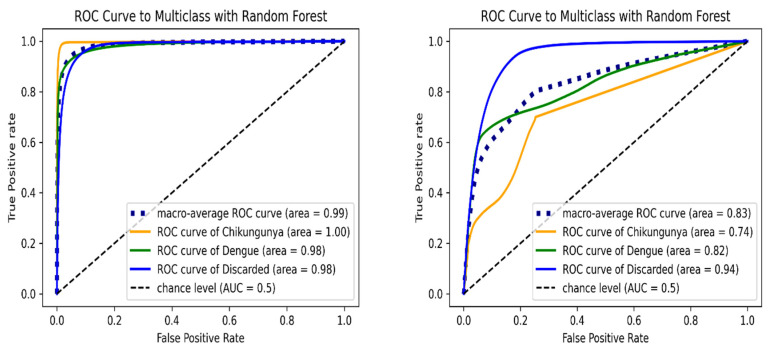
ROC curve of multi-class classification using Random Forest model on the validation (**left**) and internal test set (**right**). ROC curves are indicated in orange (chikungunya), green (dengue), blue (discarded) and blue dashed (macro-average) lines.

Figure 3 shows the ROC curve of RF algorithm for each class. In the validation test set, the macro-average AUC was 0.9865, and chikungunya class had the maximum AUC value. For the internal test set, the RF model achieved a lower overall AUC of 0.8329; the highest AUC was observed in discarded class. The RF model presented similar AUC values of discarded class in both the validation and external test set, illustrating the high capability of the proposed algorithm in predicting discarded cases.

### 3.3. The Performance of DL Model

Table 5 illustrates the proposed DL model which achieved 0.8984 macro-average specificity, 0.8401 recall/balanced accuracy, and 0.8693 AUC. The dengue class achieved the best specificity (0.9879), precision (0.9283), and F1-score (0.8297). The chikungunya class achieved the best recall (0.9986), balanced accuracy (0.9283), and AUC (0.9283). These results demonstrated that the ANN model worked best as a screening tool for chikungunya cases.

**Figure 4:**
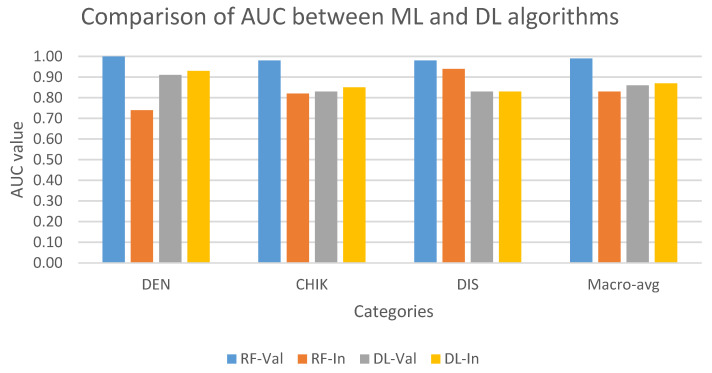
Comparison of AUC values on multi-class classification task between ML and DL models on the validation and internal test set. AUC values are indicated in blue (RF-Val: validation set using Random Forest), orange (RF-In: internal test set using Random Forest), gray (DL-Val: validation set using Deep Learning), and yellow (DL-In: internal test set using Deep Learning) bar. (Notes: DEN: dengue class; CHIK: chikungunya class; DIS: discarded class).

Figure 4 compares the performance of multi-class models on the validation and internal test set. In validation set, the AUC of ML model (RF) was higher than the DL model (ANN) in all three classes. In contrast, except for the discarded class, the ANN model performed better RF in classifying dengue and chikungunya classes in the internal test set. In addition, RF showed the best result in predicting discarded class with the approximate AUC values in the validation and internal test set, while the ANN model showed a stable performance in both datasets in all three classes.

## 4. Discussion

In this study, we investigated different ML and ANN models in differentiating dengue and chikungunya with the discarded cases. Our work suggested that the RF model works best in differentiating dengue and chikungunya with a macro-averaged recall of 0.92288, precision of 0.9111, and F1-score of 0.9196 which are higher than the metrics of a previous work using the GB model on the balanced dataset and achieved the recall, precision, and F1-score of 0.6257, 0.6205, and 0.6196, respectively [34]. Previous studies also indicated tree-based ML algorithms, such as DT and RF, achieved better performance in the multi-class classification of target variable [33,34]. A recent study also agreed that a tree-based ML model could be a suitable choice for building a decision-making support application and deploying on portable devices to assist doctors and nurses in diagnosing dengue patients [41].

Some clinical symptoms that are declared as important in dengue diagnosis were absent in the input data used for training the models. For instance, abdominal pain and myalgia were identified as better predictors of dengue infections with logistic regression and decision-tree models [36]. Some comorbidities, like pre-existing renal disease or diabetes, were ignored by the models although these symptoms are important risk factors of SD [42]. Diabetes is also associated with an increased risk for severe outcomes in dengue and West Nile fever [43].

Moreover, our study exclusively used demographic- and clinical data to train the models, which achieved a high performance. From a clinical perspectives, epidemiological and demographic variables are perceived as less influential and are usually ignored when diagnosing patients with dengue or chikungunya. In a previous study, experienced physicians only selected clinical symptoms, two pre-existing diseases (diabetes and hypertension), and days from symptoms onset as input data for training ML models [34]. Prior investigations [34,41], along with our study, indicate that epidemiological and demographic data, such as gender, age, indigenous status, and epidemiological week-of-symptoms onset, are important features for differentiating diagnosis of dengue with other arboviral diseases. For chikungunya, Vidal et al. reported gender differences in virus infection, where chikungunya symptoms are more frequent in women than men [44], these results are similar to our study’s findings. Researchers also observed that male patients confirmed to have dengue required longer recovery time compared to female patients, and patient age has a significant positive correlation with the number of clinical symptoms [45].

Our study has some advantages: First, we utilized a dataset of more than one million records as input, representing the largest dataset to date used for the multi-class classification task of dengue and chikungunya. Dataset size can affect model performance, as a larger input generally enhances the accuracy of both ML and DL algorithms [46]. Second, the input data demonstrated a high imbalance across target categories, similar to the real-world conditions while two previous studies only used the same dataset with the balanced records of each category in the target feature [34,47]. Lastly, the use of internal test sets for evaluating performance of proposed algorithms is a specific approach. To the best of our knowledge, this is the first study to incorporate internal test set to evaluate model performance for arboviral diseases.

The use of an internal test set will enhance the reliability of ML and DL algorithms when predicting data not previously encountered When deployed through computer interfaces or portable devices, these models can assist frontline healthcare workers by providing accurate and timely differentiation of arboviral diseases such as dengue and chikungunya. For instance, a young physician in remote areas of one province can use the model, enter epidemiological and clinical information of a new patient who comes from another province, and achieve a reliable diagnosis of that patient to plan medical assistance for him/her, such as hospitalization. This model could be trained with other arboviral diseases like Zika or yellow fever for active disease-surveillance and case-management in the field.

However, this study has some limitations. First, our data did not have some important features, such as the number of days from the symptom onset, one of critical criteria for deciding which laboratory test will be used. If symptom onset is less than 5 days, the NS1 antigen test kit may be preferred, whereas IgM will be used after day 5 of onset [18]. Another concerns the approach used to address the imbalanced dataset. We only applied the SMOTE technique, which made it difficult to compare the models’ performance with previous studies that either used balanced data or applied other methods like down sampling technique. In addition, the lack of interpretability analysis of top prediction features for clinical adoption is another limitation of this study, and we hope to apply SHAP analysis in future work to understand more which features contribute most to disease prediction.

## 5. Conclusions

In this study, we developed a multi-class classification method for predicting dengue and chikungunya diseases using ML and DL models. Our proposed models showed promising results as a decision-making support system to assist health physicians in differentiating dengue from chikungunya and inconclusive cases with high sensitivity, particularly in settings where laboratory testing is not readily accessible. By incorporating an internal test set, these models might have a potential application as a supportive tool in screening dengue and chikungunya diseases in Brazilian populations. Future work will continue to enhance the capability of these algorithms to differentiate more arboviral diseases, such as Zika, yellow fever, and West Nile fever diseases.

## Figures and Tables

**Table 1:** Hyperparameters with the best results for each model.

Model	Hyperparameter	Value
RF	n_estimators	100
	criterion	gini
DT	criterion	gini
AD	n_estimators	50
	learning_rate	0.1
GB	loss	log_loss
	n_estimators	100
	learning_rate	0.1
XG	objective	multi:softprob
	num_class	3
	n_estimators	100
	learning_rate	0.1
KNN	n_neighbors	3

**Table 2:** The model configuration of the proposed ANN algorithm.

Layer (Type)	Output Shape	Parameters
dense_31 (Dense)	(None, 64)	1984
dropout_9 (Dropout)	(None, 64)	0
dense_32 (Dense)	(None, 64)	4160
dropout_10 (Dropout)	(None, 64)	0
dense_33 (Dense)	(None, 64)	4160
dropout_11 (Dropout)	(None, 64)	0
dense_34 (Dense)	(None, 3)	195

Total params: 10,499; trainable params: 10,499; non-trainable params: 0.

**Table 3:** Data characteristics of features.

Features	Dengue (*n* = 4,307,513)	Chikungunya (*n* = 325,000)	Discarded (*n* = 2,100,029)	Total (*n* = 6,732,542)
Demographic data				
Gender, (%)				
Women	2,403,184 (55.8)	194,780 (59.9)	1,133,495 (54.0)	3,731,577 (55.4)
Men	1,904,329 (44.2)	130,220 (40.1)	966,534 (46.0)	3,000,965 (44.6)
Age, mean (SD)	33 (18)	37 (20)	31 (18)	32 (18)
Race, (%)				
White	1,200,564 (27.9)	39,443 (12.1)	600,871 (28.6)	1,840,878 (27.3)
Black	155,374 (3.6)	14,505 (4.5)	73,794 (3.5)	243,673 (3.6)
Yellow	30,124 (0.7)	3998 (1.2)	14,018 (0.7)	48,140 (0.7)
Brown	1,341,361 (31.1)	170,074 (52.3)	765,733 (36.5)	2,277,168 (33.8)
Indigenous	10,246 (0.2)	691 (0.2)	4547 (0.2)	15,484 (0.2)
Missing/Ignored	1,569,844 (36.5)	96,289 (29.7)	641,066 (30.5)	2,307,199 (34.3)
Pregnant, (%)				
1st Quarter	7915 (0.2)	910 (0.3)	4816 (0.2)	13,641 (0.2)
2nd Quarter	10,007 (0.2)	1505 (0.5)	5951 (0.3)	17,463 (0.3)
3rd Quarter	7951 (0.2)	1204 (0.4)	5068 (0.2)	14,223 (0.2)
Missing/Ignored	4,281,640 (99.4)	321,381 (98.8)	2,084,194 (99.3)	6,687,215 (99.3)
Educational Degree, (%)				
Elementary School	229,742 (5.3)	15,434 (4.8)	116,632 (5.6)	361,808 (5.4)
Middle School	406,366 (9.5)	24,394 (7.5)	200,904 (9.6)	631,664 (9.4)
High School	698,230 (16.2)	37,686 (11.6)	357,369 (17.0)	1,093,285 (16.2)
College	168,808 (3.9)	8495 (2.6)	88,610 (4.2)	265,913 (3.9)
Missing/Ignored	2,804,367 (65.1)	238,991 (73.5)	1,336,514 (63.6)	4,379,872 (65.1)
Clinical data				
Fever, (%)	1,714,334 (39.8)	139 (<0.1)	793,551 (37.8)	2,508,024 (37.3)
Myalgia, (%)	1,595,876 (37)	117 (<0.1)	693,411 (33)	2,289,404 (34)
Headache, (%)	1,611,029 (37.4)	115 (<0.1)	714,290 (34)	2,325,434 (34.5)
Rash, (%)	466,788 (10.8)	49 (<0.1)	154,211 (7.3)	621,048 (9.2)
Vomit, (%)	438,160 (10.2)	42 (<0.1)	194,662 (9.3)	632,864 (9.4)
Nausea, (%)	691,305 (16)	58 (<0.1)	267,463 (12.7)	958,826 (14.2)
Back pain, (%)	545,952 (12.7)	54 (<0.1)	208,859 (9.9)	754,865 (11.2)
Conjunctivitis, (%)	64,807 (1.5)	13 (<0.1)	25,708 (1.2)	90,528 (1.3)
Arthritis, (%)	214,337 (5)	30 (<0.1)	73,742 (3.5)	288,109 (4.3)
Arthralgia, (%)	451,362 (10.5)	58 (<0.1)	183,955 (8.8)	635,375 (9.4)
Petechiae, (%)	187,214 (4.3)	26 (<0.1)	58,980 (2.8)	246,220 (3.7)
Tourniquet test, (%)	97,642 (2.3)	5 (<0.1)	22,189 (1.1)	119,836 (1.8)
Retro-orbital pain, (%)	730,885 (17)	46 (<0.1)	231,113 (11)	962,044 (14.3)
Comorbidity data				
Diabetes, (%)	45,088 (1)	8 (<0.1)	18,561 (0.9)	63,657 (0.9)
Hematological disease, (%)	8751 (0.2)	1 (<0.1)	3949 (0.2)	12,701 (0.2)
Liver disease, (%)	9351 (0.2)	1 (<0.1)	4243 (0.2)	13,595 (0.2)
Kidney disease, (%)	7920 (0.2)	1 (<0.1)	3390 (0.2)	11,311 (0.2)
Hypertension, (%)	112,685 (2.6)	12 (<0.1)	44,082 (2.1)	156,779 (2.3)
Peptic acid disease, (%)	10,258 (0.2)	2 (<0.1)	4582 (0.2)	14,842 (0.2)
Autoimmune disease, (%)	8031 (0.2)	0 (0)	3287 (0.2)	11,318 (0.2)

**Table 4:** The performance of different ML models.

ML Models	Accuracy	Specificity	Recall	Precision	F1-Score	AUC
Without SMOTE						
RF	0.8501	0.8994	0.9016	0.7846	0.8245	0.9436
DT	0.8384	0.8884	0.8785	0.7858	0.8226	0.9225
AD	0.7240	0.8376	0.6581	0.5974	0.5919	0.8414
GB	0.8601	0.9452	0.9068	0.7946	0.8358	0.9576
XG	0.9113	0.9351	0.9333	0.8711	0.8983	0.9831
KNN	0.8641	0.9064	0.9104	0.8059	0.8443	0.9686
With SMOTE						
RF	0.9292	0.9562	0.9288	0.9111	0.9196	0.9853
DT	0.9221	0.9470	0.9137	0.9072	0.9104	0.9347
AD	0.8338	0.9033	0.8703	0.7776	0.8147	0.9101
GB	0.8664	0.9329	0.9101	0.8061	0.8452	0.9675
XG	0.9141	0.9531	0.9357	0.8733	0.9007	0.9841
KNN	0.9248	0.9561	0.9342	0.8907	0.9108	0.9748

(Note: Machine learning (ML), Random Forest (RF), Decision Tree (DT), Adaptive Boosting (AD), Gradient Boosting Machine (GB), eXtreme Gradient Boosting (XG), K-nearest Neighbor (KNN)).

**Table 5:** Proposed DL model’s performance.

Class	Accuracy	Specificity	Recall	Balanced Accuracy	Precision	F1-Score	AUC
Chikungunya	0.8648	0.8580	0.9986	0.9283	0.2628	0.4161	0.9283
Dengue	0.8125	0.9879	0.7138	0.8509	0.9906	0.8297	0.8509
Discarded	0.8364	0.8494	0.8078	0.8286	0.7083	0.7548	0.8286
Macro-average	0.7568	0.8984	0.8401	0.8401	0.6539	0.6669	0.8693

## Data Availability

All data used in this study are publicly available and were obtained from the open-access dataset shared by da Silva Neto et al. (2022) [37] in Scientific Data [https://www.nature.com/articles/s41597-022-01312-7] (accessed on 1 June 2025). The dataset includes anonymized epidemiological records of dengue and chikungunya cases in Brazil from 2013 to 2020. No additional restrictions apply to the use of this dataset.

## Abbreviations

The following abbreviations are used in this manuscript:

AD	Adaptative boosting
AI	Artificial intelligence
ANN	Artificial neural network
AUC	Area under the curve
DF	Dengue fever
DHF	Dengue hemorrhagic fever
DL	Deep learning
DNN	Deep neural network
DT	Decision tree
ETC	Extra tree classifier
FP	False positive
FN	False negative
GB	Gradient boosting machine
KNN	k-nearest neighbor
LR	Logistic regression
ML	Machine learning
NB	Naïve Bayes
NTDs	Neglected tropical diseases
RF	Random forest
RFECV	Recursive Feature Elimination with Cross-Validation
ROC	Receiver operating characteristics
SD	Severe dengue
SMO	Sequential minimal optimization
SMOTE	Synthetic minority oversampling technique
SVC	Support vector classifier
SVM	Support vector machine
TN	True negative
TP	True positive
WHO	World Health Organization
XG	eXtreme gradient boosting
